# Electronic Warfare Target Location Methods, Second Edition. Edited by Richard A. Poisel, Artech House, 2012; 422 pages. Price: £99.00, ISBN 978-1-60807-523-2

**DOI:** 10.3390/s130101151

**Published:** 2013-01-17

**Authors:** Shu-Kun Lin

**Affiliations:** MDPI AG, Kandererstrasse 25, CH-4057 Basel, Switzerland; E-Mail: lin@mdpi.com

The following paragraphs are reproduced from the website of the publisher [1].

Describing the mathematical development underlying current and classical methods of geolocating electronic systems that are emitting, this newly revised and greatly expanded edition of a classic Artech House book offers practical guidance in electronic warfare target location. The Second Edition features a wealth of additional material including new chapters on time delay estimation, direction finding techniques, and the MUSIC algorithm. This practical resource provides you with critical design information on geolocation algorithms, and establishes the fundamentals of existing algorithms as a launch point for further algorithm development. You gain an in-depth understanding of key target location methods that you can effectively apply to your work in the field. You discover triangulation algorithms that offer a highly efficient way to geolocate targets when the real estate on the sensor systems is adequate to support an antenna array. The book also presents quadratic geolocation techniques that can be implemented with extremely modest antennas—frequently a single dipole or monopole. Moreover, this authoritative volume details methods for geolocating the source of high frequency signals with a single sensor site.

**Table of Contents****Preface xv****Chapter 1 Introduction to Emitter Geolocation**1.1Introduction1.2Gradient Descent Algorithm1.3Concluding RemarksReferences**Chapter 2 Triangulation**2.1Introduction2.2Basic Concepts2.3Least-Squares Error Estimation2.4Total Least-Squares Estimation2.5Least-Squares Distance Error PF Algorithm2.5.1Brown's Least-Squares Triangulation Algorithm2.5.2Hemispheric Least-Squares Error Estimation Algorithm2.5.3Pages-Zamora Least-Squares2.5.4Total Least-Squares Error2.6Minimum Mean-Squares Error Estimation2.6.1Dynamical Systems2.6.2Linear Minimum Mean-Squares Estimation2.6.3Target Location Estimation with the Linear Model2.6.4Kalman Filter Methods2.7The Discrete Probability Density Method2.8Generalized Bearings2.9Maximum Likelihood PF Algorithm2.9.1Maximum Likelihood Estimation Triangulation Algorithm2.9.2Maximum Likelihood Estimation Algorithm Comparison2.10Multiple Sample Correlation2.11Bearing-Only Target Motion Analysis2.12Sources of Error in Triangulation2.12.1Geometric Dilution of Precision in Triangulation2.12.2LOB Error2.12.3Effects of Bias on Bearing-Only PF2.12.4Combining Noisy LOB Measurements2.12.5Effects of Navigation Error2.13Concluding RemarksReferencesAppendix 2A Least-Squares Error Estimation Program ListingAppendix 2B Generalized Bearing Program Listing**Chapter 3 DF Techniques**3.1Introduction3.2Array Processing Direction of Arrival Measurement Methods3.2.1Introduction3.2.2The Model3.2.3Array Covariance Modeling3.2.4Direction of Arrival3.2.5Subspace-Based Methods3.2.6Beamforming AOA Estimation3.2.7Maximum Likelihood AOA Estimation3.2.8Least-Square Error AOA Estimation3.2.9Decoupling Sample Source Signals from AOA Parameters3.2.10Gram-Schmidt Orthogonalization3.2.11Nonlinear Programming3.3Other Methods of Estimating the AOA3.3.1Phase Interferometry3.3.2Amplitude Systems3.3.3Doppler Direction Finder3.4MSE Phase Interferometer3.4.1Introduction3.4.2The Algorithm3.4.3Simulation3.5DF with a Butler Matrix3.5.1Introduction3.5.2Beamforming Network3.5.3Summary3.6Phase Difference Estimation Using SAW Devices3.6.1Introduction3.6.2SAW Characteristics3.7Concluding RemarksReferences**Chapter 4 MUSIC**4.1Introduction4.2MUSIC Overview4.3MUSIC4.3.1The MUSIC Algorithm4.4Performance of MUSIC in the Presence of Modeling Errors4.4.1Model Errors4.4.2Error Expressions4.4.3Results4.5Determining the Number of Wavefields4.6Effect of Phase Errors on the Accuracy of MUSIC4.6.1Introduction4.6.2Accuracy4.6.3Solutions for Errors4.6.4Statistics4.6.5Horizontal Planar Arrays4.6.6Simulations4.6.7Summary4.7Other Superresolution Algorithms4.7.1Maximum Likelihood Method4.7.2Adaptive Angular Response4.7.3Thermal Noise Algorithm4.7.4Maximum Entropy Method4.7.5Comparisons4.8Concluding RemarksReferences**Chapter 5 Quadratic Position-Fixing Methods**5.1Introduction5.2TDOA Position-Fixing Techniques5.2.1Introduction5.2.2TDOA5.2.3Calculating the PF with TDOAs5.2.4Nonlinear Least-Squares5.2.5TDOA Measurement Accuracy5.2.6TDOA PFs with Noisy Measurements5.2.7TDOA Dilution of Precision5.2.8Bias Effects of TDOA PF Estimation5.2.9Effects of Movement on TDOA PF Estimation5.3Differential Doppler5.3.1Introduction5.3.2DD5.3.3DD Measurement Accuracy5.3.4Maximum Likelihood DD Algorithms5.3.5Cross-Ambiguity Function5.3.6Estimating the DD of a Sinusoid in Noise Using Phase Data5.3.7Effects of Motion on DD PF Estimating5.4Range Difference Methods5.4.1Introduction5.4.2Least-Squared Range Difference Methods5.4.3Range Difference Using Feasible Bivectors5.5Concluding RemarksReferences**Chapter 6 Time Delay Estimation**6.1Introduction6.2System Overview6.3Cross Correlation6.3.1Error Analysis of the Cross-Correlation Method6.3.2Flat Noise Spectra: Arbitrary Signal Spectrum6.3.3Flat Noise Spectra: Flat Signal Spectrum6.4Generalized Cross-Correlation6.5Estimating the Time Delay with the Generalized Correlation Method6.5.1Roth Process6.5.2Smoothed Coherence Transform (SCOT)6.5.3Phase Transform (PHAT)6.5.4Eckart Filter6.5.5Maximum Likelihood6.5.6Variance of the Delay Estimators6.6Time Delay Estimation Using the Phase of the Cross-Spectral Density6.6.1Introduction6.6.2Data Model6.6.3Properties of the Sample CSD6.6.4TDOA Estimation6.6.5Cramer-Rao Bound6.6.6Other Considerations6.6.7Summary6.7Effects of Frequency and Phase Errors in EW TDOA Direction-Finding Systems6.7.1Introduction6.7.2Perfect Synchronization6.7.3Errors in Synchronization6.7.4Effects of Finite Sample Time6.7.5White Noise Signal6.7.6Simulation Results6.7.7Estimator Performance6.7.8Ramifications6.7.9Summary6.8Concluding RemarksReferences**Chapter 7 Single-Site Location Techniques**7.1Introduction7.2HF Signal Propagation7.2.1Ionograms7.2.2Magnetic Field Effects7.3Single-Site Location7.4Passive SSL7.5Determining the Reflection Delay with the Cepstrum7.6MUSIC Cepstrum SSL7.7Earth Curvature7.8Skywave DF Errors7.8.1Introduction7.8.2Magnetic Field Effects7.8.3Ross Curve7.8.4Bailey Curve7.9Ray Tracing7.9.1Parabolic Modeling7.10Accuracy Comparison of SSL and Triangulation for Ionospherically Propagated Signals7.10.1Introduction7.10.2Spherical Model7.10.3Plane Model7.10.4Comparison Between SSL and Triangulation7.10.5Summary7.11Concluding RemarksReferences**Appendix A Grassmann Algebra**A.1BackgroundA.2IntroductionA.3Exterior ProductA.3.1Properties of the Exterior ProductA.3.2m-VectorsA.4Regressive ProductA.4.1Unions and Intersections of SpacesA.4.2Properties of the Regressive ProductA.4.3The Common Factor AxiomA.4.4The Common Factor TheoremA.4.5The Intersection of Two Bivectors in a 3-D SpaceA.5Geometric InterpretationsA.5.1Points and VectorsA.5.2Sums and Differences of PointsA.5.3Lines and PlanesA.5.4Intersection of Two LinesA.6The ComplementA.6.1The Complement as a Correspondence Between SpacesA.6.2The Euclidean ComplementA.6.3The Complement of a ComplementA.6.4The Complement AxiomA.7The Interior ProductA.7.1Inner Products and Scalar ProductsA.7.2Calculating Interior ProductsA.7.3Expanding Interior ProductsA.7.4The Interior Product of a Bivector and a VectorA.8Concluding RemarksReferences**Appendix B Nonlinear Programming Algorithms**B.1IntroductionB.2Steepest DescentB.2.1IntroductionB.2.2Method of Steepest DescentB.2.3ConvergenceB.2.4ScalingB.2.5ExtensionsB.3Gauss-Newton MethodB.4Levenberg-Marquardt AlgorithmB.4.1IntroductionB.4.2Nonlinear Least-Squares MinimizationB.4.3LM as a Blend of Gradient Descent and Gauss-Newton IterationB.5Concluding Remarks**References****Acronyms****Symbols****About the Author****Index**
